# Associations of inflammatory biomarkers with clinical outcomes in degenerative lumbar spinal stenosis: A systematic review

**DOI:** 10.1016/j.bas.2026.106056

**Published:** 2026-04-18

**Authors:** Michita Noma, Niek Djuric, Carmen Vleggeert-Lankamp

**Affiliations:** a Department of Neurosurgery, Leiden University Medical Center, Albinusdreef 2, Leiden, 2333 ZA, Netherlands; b Department of Orthopedic Surgery, The University of Tokyo Hospital, 7-3-1 Hongo, Bunkyo-ku, Tokyo, 113-8655, Japan; c Department of Orthopedic Surgery, Tokyo Metropolitan Hiroo General Hospital, 2-34-10, Ebisu, Shibuya-ku, Tokyo, 150-0013, Japan; d Department of Neurosurgery, Erasmus University Medical Center, Dr. Molewaterplein 40, Rotterdam, 3015 GD, Netherlands; e Department of Neurosurgery, Spaarne Gasthuis, Boerhaavelaan 22, Haarlem, 2035 RC, Netherlands

**Keywords:** Lumbar spinal stenosis, Inflammatory biomarkers, Clinical outcomes, Imaging findings

## Abstract

**Introduction:**

Inflammatory pathways have been implicated in degenerative lumbar spinal stenosis (LSS), but the clinical relevance of inflammatory biomarkers is unclear.

**Research question:**

What associations exist between inflammatory biomarkers and patient-centered outcomes in degenerative LSS, and secondarily with imaging features?

**Material and methods:**

We conducted a PRISMA 2020–compliant systematic review. MEDLINE/PubMed, Embase, Cochrane Library, Scopus, Web of Science, CINAHL, and Academic Search Premier were searched from inception to July 4, 2025. We included original adult human studies of degenerative LSS reporting inflammatory biomarkers (tissue, serum/plasma, or cerebrospinal fluid [CSF]) with clinical and/or imaging outcomes. Risk of bias was assessed using a modified Newcastle–Ottawa Scale.

**Results:**

Of 1157 records identified (645 from databases; 512 from citation tracking), 14 studies met inclusion criteria. Biomarker–clinical outcome evidence was sparse and based on single studies: higher CSF NOx was associated with a lower postoperative recovery rate; higher serum MCP-1 with greater short-term satisfaction after epidural steroid injection; and higher serum miR-486-5p with more pain/disability and worse postoperative JOA scores. Most studies linked biomarkers to imaging severity, particularly ligamentum flavum hypertrophy and reduced dural sac cross-sectional area. Only one small study assessed both clinical and imaging outcomes: higher CSF IL-6 associated with smaller dural sac area but not pain intensity or walking distance.

**Discussion and conclusion:**

Evidence connecting inflammatory biomarkers to patient-centered outcomes in degenerative LSS is limited. Reported associations are hypothesis-generating and require confirmation in standardized, adequately powered prospective studies before clinical application.

## Highlights

• Three biomarkers were each linked to clinical outcomes in only one study.
• Most evidence links biomarkers to imaging severity (LF hypertrophy, dural sac CSA).
• Links to both clinical and imaging outcomes were reported in only one CSF IL-6 study.
• Hypothesis-generating for prognostic and treatment-response stratification in LSS.
• Standardized, adequately powered prospective studies are needed before clinical use.

## 1 Introduction

Lumbar spinal stenosis (LSS) is a leading cause of pain, disability, and loss of independence in older adults. The classical paradigm attributes symptom generation to mechanical compression of neural elements arising from degenerative changes. Increasing evidence, however, indicates that inflammation and immune cell infiltration are integral to LSS pathogenesis and progression, potentially interacting with mechanical factors to sustain nociception and neural dysfunction (Ohtori et al., 2011; Jiang et al., 2024; Benditz et al., 2019). Observational studies have linked circulating or tissue-level inflammatory markers to imaging features such as canal narrowing, tissue fibrosis, and ligamentum flavum (LF) hypertrophy, yet the clinical implications of these processes remain uncertain. In particular, the extent to which specific inflammatory pathways relate to patient-reported pain and disability, function, quality of life, or prognosis has not been clearly defined.

Prior systematic reviews have focused largely on lumbar disc herniation and sciatica populations, in which pro-inflammatory cytokines (e.g. interleukin-6 (IL-6), tumour necrosis factor-α (TNF-α), interleukin-8 (IL-8)) correlate with higher pain intensity, whereas anti-inflammatory cytokines (e.g. interleukin-4 (IL-4), interleukin-10 (IL-10)) may be protective (Jungen et al., 2019; Djuric et al., 2020). In particular, Djuric et al. systematically synthesized associations between M1-and M2-related cytokines and visual analog scale (VAS) pain scores in patients with lumbar disc herniation, suggesting that macrophage-related inflammatory profiles may be linked to symptom severity (Djuric et al., 2020). These syntheses, however, did not specifically address LSS due to degenerative causes. A recent review by Mualem et al. (2024) summarized associations between inflammatory biomarkers and LF hypertrophy in LSS, but did not systematically evaluate clinical relevance or broader radiological correlations (Mualem et al., 2024).

To address these gaps, we conducted a systematic review focused on symptomatic degenerative LSS to evaluate associations between inflammatory biomarkers—measured in tissue, serum, or cerebrospinal fluid (CSF)—and clinically meaningful outcomes (pain, disability, function, quality of life, and prognosis). As a secondary objective, we examined relationships between these biomarkers and imaging features. Wherever possible, we further contextualized these associations within an exploratory macrophage polarization (M1/M2) framework to provide biological context for candidate biomarkers in degenerative LSS. Compared with related spinal disorders such as lumbar disc herniation, however, macrophage polarization has been less directly studied in degenerative LSS, highlighting an important gap in the current literature.

## 2 Methods

### 2.1 Protocol and eligibility criteria

This systematic review was conducted and reported in accordance with the Preferred Reporting Items for Systematic Reviews and Meta-Analyses (PRISMA) 2020 statement. We included original human studies of adults (≥18 years) with degenerative LSS defined by compatible symptoms and confirmed on magnetic resonance imaging (MRI) or computed tomography (CT). Studies enrolling mixed pathologies (e.g., LSS plus disc herniation) were eligible only when LSS-specific results were reported separately. The exposures of interest were inflammatory biomarkers, broadly encompassing cellular and molecular components of inflammation—immune-cell infiltration and soluble mediators (e.g., cytokines, chemokines)—assessed in tissue, serum/plasma, or CSF. Comparators could include healthy controls, lower disease-severity strata, or groups with lower biomarker levels/inflammatory activity. Primary outcomes were patient-centered measures (pain intensity, functional disability, health-related quality of life). Secondary outcomes were imaging features as defined by each study. We excluded animal/in vitro studies, case reports, narrative reviews, editorials/letters, conference abstracts, non-English publications, and studies concerning non-degenerative causes of stenosis (trauma, infection, neoplasm, congenital).

### 2.2 Information sources and search strategy

We developed a comprehensive strategy that combined controlled vocabulary and free-text terms for LSS, inflammatory biomarkers, and biospecimen sources (e.g., “lumbar spinal stenosis,” “biomarker,” “cytokine,” “ligamentum flavum,” “serum,” “cerebrospinal fluid”). We searched MEDLINE/PubMed, Embase, Cochrane Library, Scopus, Web of Science, Cumulative Index to Nursing and Allied Health Literature (CINAHL), and Academic Search Premier, from inception to 4 July 2025. The full strategies of search strings are provided in the Supplementary Material. Afterwards, we screened the reference lists of the included articles and related articles to identify potentially relevant reports.

### 2.3 Study selection and risk of bias assessment

Two reviewers independently screened titles/abstracts against prespecified criteria, obtained full texts for potentially eligible reports, and made final inclusion decisions. Disagreements were resolved by discussion to reach consensus.

Study-level risk of bias was appraised with a Newcastle-Ottawa Scale (NOS) for observational studies. Two reviewers independently assigned points for each domain (selection, comparability, and outcome/exposure). We defined studies with 7–9 points as having a low risk of bias, those with 4–6 points as moderate risk, and those with 0–3 points as high risk. Any disagreements were resolved through discussion until consensus was achieved.

### 2.4 Synthesis approach

Given the anticipated and observed heterogeneity in biomarker classes, biospecimen matrices, assay methods, and clinical instruments, we did not perform a quantitative meta-analysis. Instead, we conducted a structured narrative synthesis.

## 3 Results

### 3.1 Study selection

**Fig. 1:**
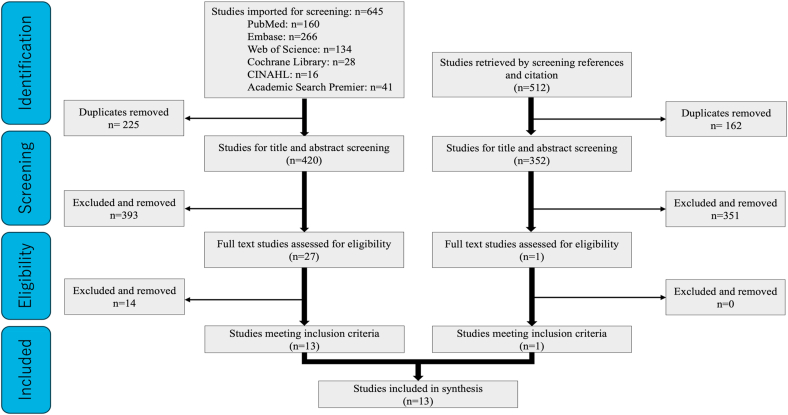
Flowchart of the study selection. The database search identified 645 records; after removing 225 duplicates, 420 records were screened. Of these, 27 full texts were assessed and 13 studies met the inclusion criteria. Subsequently, citation/reference tracking identified 512 additional records; after removing 162 duplicates, 352 titles/abstracts were screened, 1 proceeded to full-text and 1 met the inclusion criteria. In total, 14 studies were included in the synthesis.

Database searches (PubMed, 160; Embase, 266; Web of Science, 134; Cochrane Library, 28; CINAHL, 16; Academic Search Premier, 41) identified 645 records. After deduplication, 420 unique records remained. Title and abstract screening retained 27 reports for full-text assessment, of which 13 met the eligibility criteria. Backward citation searching of these 13 articles yielded 512 references; after removing duplicates, 352 unique references were screened, and 1 additional study was included. In total, 14 studies were included (Fig. 1) and underwent risk-of-bias assessment.

### 3.2 Risk of bias within studies

Of the 14 included studies, seven were rated as having a low risk of bias and seven a moderate risk; none were rated as high risk. In the selection domain, scores were generally favorable because biomarker exposure and outcome measures were clearly defined and consistently ascertained across studies. Representativeness of the study cohort was adequate in 7/14 studies, and 5 studies did not include a control group. In the comparability domain, 12/14 studies adjusted for potential confounders. Outcome assessment received full scores in 12/14 studies, and only one study lacked adequate follow-up. Overall, potential bias was mainly related to cohort representativeness and comparability, whereas outcome assessment was consistently strong.

### 3.3 Overview of included studies

Study designs comprised 11 cross-sectional case–control studies and 3 observational cohorts, including 1 prospective cohort. Table 1 summarizes, for each study, the design, clinical and imaging outcomes, and the direction of association between individual biomarkers and outcomes. Across studies, outcomes assessed were clinical measures in 3 studies, combined clinical and imaging measures in 1 study, and imaging measures alone in 10 studies. Clinical outcomes included visual analog scale (VAS) pain scores, Oswestry Disability Index (ODI) and Japanese Orthopaedic Association (JOA) scores for disability, and patient satisfaction. Imaging outcomes included LF thickness and spinal canal stenosis quantified as dural sac cross-sectional area (CSA). Among cohort studies, follow-up ranged from 2 to 6 months.

**Table 1:** Characteristics and key findings of included studies.

Outcome Type	Authors & Year	Journal	Country	Study Design	Patient Characteristics	Biomarker	Specimen/Modality	Assay	Clinical Outcome/Imaging Characteristics	Key Findings
Clinical	(Denda et al., 2011)	European Spine Journal	Japan	Observational cohort	LSS n = 70; Controls n = 53	Nitric oxide metabolites (NOx)	CSF	Griess method	Pre/post-op JOA; post-op Hirabayashi recovery rate	NOx in CSF higher in LSS vs controls; NOx negatively correlated with post-op Hirabayashi recovery rate(r = −0.24, p < 0.05); not correlated with pre/post-op JOA.
Clinical	(Lin et al., 2020)	PM&R	USA	Prospective observational cohort	LSS undergoing epidural steroid injection n = 11	MCP-1	Serum	Immuno-sorbent assay	SSSQ Satisfaction at 2 months	Higher baseline MCP-1 in serum was correlated with better 2-month SSSQ satisfaction(r = −0.915, P = 0.03).
Clinical	(Zhang et al., 2024)	European Spine Journal	China	Observational cohort	LSS n = 52; LDH n = 46; Healthy n = 42	miR-486-5p	Serum	qPCR for miRNA; ELISA for cytokines	VAS, ODI, JOA at 6 months	miR-486-5p in serum was higher in LSS than controls and was positively correlated with VAS(r = 0.614, P < 0.001) and ODI(r = 0.665, P < 0.001) and negatively with JOA(r = −0.651, P < 0.001)—worse 6-month outcomes; levels declined after surgery.
Clinical/Imaging	(Ohtori et al., 2011)	European Spine Journal	Japan	Cross-sectional case–control	LSS n = 30; Controls n = 10	IL-6	CSF; MRI	ELISA	VAS, walking distance; dural sac CSA	IL-6 in CSF was higher in LSS than controls and positively correlated with MRI stenosis severity(r = 0.81, p = 0.01) and negatively with dural sac CSA(r = - 0.8, p = 0.01), and not correlated with pain scores or walking distance.
Imaging	(Nakamura et al., 2015)	European Spine Journal	Japan	Cross-sectional case–control	LSS n = 35; non-LSS n = 15	IL-6	LF tissue; MRI	qPCR, IHC, ELISA	MRI LF thickness	LF IL-6 mRNA expression was positively correlated with LF thickness(r = 0.40, p < 0.01).
Imaging	(Torun et al., 2023)	Asian Spine Journal	Turkey	Cross-sectional case–control	LSS n = 23; LDH n = 12	DDR1, DDR2; MMP-13	LF tissue; MRI	qRT-PCR, Western blot, histology	MRI LF thickness	LF DDR1/2 and MMP-13 expression were positively correlated with LF thickness(r = 0.408, p = 0.015; r = 0.361, p = 0.033; r = 0.341, p = 0.045; respectively).
Imaging	(Yamahata et al., 2017)	Journal of Orthopaedic Science	Japan	Cross-sectional case–control	LSS n = 10; non-LSS controls n = 10	JAK/STAT pathway (p-STAT3)	LF tissue; MRI	Western blot, IHC	MRI LF thickness	The proportion of p-STAT3 positive cells was positively correlated with LF thickness(r = 0.63, p = 0.01).
Imaging	(Oh et al., 2013)	Connective Tissue Research	Korea	Cross-sectional case–control	LSS n = 10; Non-degenerative controls n = 11	CX3CL1/CX3CR1; serum sFKN	LF tissue; serum; MRI	qPCR, IHC, ELISA	MRI LF thickness	Tissue CX3CL1/CX3CR1 and sFKN in serum positively correlated with LF thickness(r = 0.819, p = 0.000; r = 0.716, p = 0.001; r = 0.907, p = 0.003; respectively).
Imaging	(Lakemeier et al., 2012)	Acta Neurochirurgica	Germany	Cross-sectional case–control	LSS n = 38; LDH controls n = 12	CD44, CD44v5, CD44v6	LF tissue; MRI	IHC	MRI LF thickness; dural sac CSA	CD44 and CD44v5 expression increased in LSS and positively correlated with LF thickness and negatively with dural sac CSA(r is not mentioned, p < 0.05). CD44v6 showed no correlation.
Imaging	(Zhang et al., 2010)	Spine	China	Cross-sectional case–control	LSS n = 10; LDH n = 10	PDGF-BB	LF tissue; MRI	IHC, qPCR, Western blot	MRI LF thickness	PDGF-BB higher in LSS; positively correlated with LF thickness(r = 0.41, p < 0.05).
Imaging	(Sun et al., 2018)	Bioscience Reports	China	Cross-sectional case–control	LSCS n = 12; LDH n = 12	Leptin	LF tissue; MRI	IHC, qPCR, Western blot	MRI LF thickness	Leptin mRNA expression in LF positively correlated with LF thickness(r = 0.799, P < 0.05).
Imaging	(Cui et al., 2011)	Journal of Orthopaedic Science	Japan	Cross-sectional case–control	LSCS with diabetes n = 11; without n = 24	MMP-13	LF tissue; CT	qRT-PCR; histology	CT LF thickness	MMP-13 was positively correlated with LF thickness(r = 0.43, P = 0.01).
Imaging	(Sugimoto et al., 2018)	PLOS ONE	Japan	Cross-sectional case–control	LSCS n = 31; Controls n = 21	MMP-2/9	LF tissue; MRI	qPCR, ELISA, Western blot, IHC	MRI LF thickness	MMP-2 mRNA and protein expression were positively correlated with LF thickness(r = 0.643, p < 0.01; r = 0.699, p < 0.01); whereas MMP-9 showed no correlation.
Imaging	(Hur et al., 2015)	Neurosurgery	Korea	Cross-sectional case–control	LSCS n = 20; Controls n = 16	VEGF, Angopoietin-1, VCAM; CD34^+^ microvessel density	LF tissue; MRI, X-ray	ELISA; IHC for CD34^+^	MRI LF thickness; X-ray segmental range of motion	VEGF expression and CD34^+^ microvessel density were positively correlated with LF thickness(r = 0.557, P = 0.001), segmental motion(r = 0.586, P = 0.001); angiopoietin-1 and VCAM showed no correlation.
	

Abbreviation: cluster of differentiation 34–positive (CD34^+^), computed tomography (CT), cross-sectional area (CSA), discoidin domain receptor (DDR), enzyme-linked immunosorbent assay (ELISA), fractalkine (FKN), immunohistochemistry (IHC), interleukin-6 (IL-6), Janus kinase/signal transducer and activator of transcription (JAK/STAT), Japanese Orthopaedic Association (JOA), ligamentum flavum (LF), magnetic resonance imaging (MRI), matrix metalloproteinase (MMP), monocyte chemoattractant protein-1 (MCP-1), nitric oxide metabolites (NOx), Oswestry Disability Index (ODI),pain disability questionnaire (PDQ), platelet-derived growth factor-BB (PDGF-BB), phosphorylated signal transducer and activator of transcription 3 (pSTAT3), quantitative polymerase chain reaction (qPCR), Swiss Spinal Stenosis Questionnaire (SSSQ), vascular endothelial growth factor (VEGF), visual analog scale (VAS).

Specimens and imaging modalities varied: analyses of LF tissue plus MRI were most frequent (n = 7), followed by serum-based assessments (n = 2), CSF-based assessments (n = 1), CSF plus MRI (n = 1), LF tissue plus serum plus MRI (n = 1), LF tissue plus CT (n = 1), and LF tissue plus MRI/plain radiography (n = 1). Geographically, six studies were conducted in Japan, three in China, two in South Korea, and one each in the United States, Turkey, and Germany. Table 2 groups the investigated biomarkers into inflammatory, extracellular matrix/fibrotic, angiogenic/proliferative, metabolic/endocrine, and microRNA categories and outlines their putative roles in these processes and in macrophage polarization.

**Table 2:** Characteristics and biological relevance of each biomarker.

Category	Biomarker(s)	Potential roles in inflammation, fibrosis	Macrophage polarization
Inflammatory	NO metabolites (NOx) (Denda et al., 2011) (Rath et al., 2014)	NO synthase in macrophages and Schwann cells; excessive NO promotes neuronal injury and apoptosis, contributing to degeneration and secondary tissue remodeling.	Nitric oxide (NO), produced in large amounts by iNOS during strong M1 activation, leads to increased NOx levels, whereas NO subsequently functions as a negative-feedback mediator that restrains excessive M1 polarization. Thus, elevated NOx reflects both an M1-dominant inflammatory response and the compensatory suppression of overactivated M1 macrophages.
	MCP-1 (Lin et al., 2020) (Sierra-Filardi et al., 2014)	MCP-1 recruits monocytes and macrophages to inflamed tissue and amplifies local cytokine release.	MCP-1 recruits macrophages to inflamed tissue and can support a shift toward an M2-like, pro-resolving phenotype in M-CSF– and anti-inflammatory cytokine–rich environments; however, its effects on polarization are microenvironment-dependent and may also augment M1 programs under GM-CSF–dominant conditions.
	IL-6 (Ohtori et al., 2011) (Nakamura et al., 2015) (Sugimoto et al., 2018) (Li et al., 2022)	IL-6 supports inflammatory activation and promotes collagen synthesis and tissue remodeling.	IL-6 promotes a shift in macrophages from a pro-inflammatory M1 to a reparative M2 phenotype in fibrotic and wound-healing lesions of the skin, lung, and kidney.
	p-STAT3 (Yamahata et al., 2017) (Wang et al., 2021)	p-STAT3 reflects activation of the JAK/STAT3 pathway; its expression in endothelial and fibroblast-like cells, indicating involvement in inflammatory and fibrotic remodeling.	STAT3 signaling supports M2 macrophage polarization in liver disease.
	CX3CL1/CX3CR1; serum sFKN (Oh et al., 2013) (Gao et al., 2023)	Fractalkine (CX3CL1) exists in both membrane-bound and soluble forms. By binding to its receptor CX3CR1 on monocytes, macrophages, and fibroblast-like cells, it mediates cell adhesion and chemoattraction within chronically inflamed tissue.	CX3CL1–CX3CR1 signaling promotes M2 macrophage polarization in intervertebral disc degeneration models.
	VCAM (Hur et al., 2015) (Lin et al., 2024)	Endothelial adhesion molecule; mediates leukocyte adhesion and transmigration, linking chronic endothelial activation to sustained inflammatory cell influx and downstream fibrotic remodeling.	VCAM dependent monocyte adhesion facilitates M2 macrophage polarization in the osteosarcoma microenvironment.
Extracellular matrix/fibrotic	DDR1, DDR2, MMP-13 (Torun et al., 2023) (Soundia et al., 2024)	Collagen-activated receptors DDR1/DDR2 enhance fibroblast activity and upregulate matrix metalloproteinases—including MMP-13—thereby driving extracellular matrix remodeling and fibrosis in hypertrophied ligamentum flavum.	DDR1/DDR2 activation promotes M2-like macrophage polarization in liver fibrosis. MMP-13 is associated with M1-linked overexpression in early inflammation phase of medication-related osteonecrosis of the jaws development.
	MMP-2, MMP-9 (Torun et al., 2023) (Sugimoto et al., 2018) (Huang et al., 2012) (Yabluchanskiy et al., 2016)	MMP-2 and MMP-9 are gelatinases with elastolytic activity. MMP-2 promotes elastic fiber degradation and fibrosis, whereas MMP-9 shows limited involvement.	MMP-2 is upregulated in ligamentum flavum tissue, promoting fibrosis in an M2-associated environment. MMP-9 is increased in early inflammatory stages of LF hypertrophy, consistent with M1 macrophage–linked matrix degradation.
	CD44, CD44v5, CD44v6 (Lakemeier et al., 2012) (Zhang et al., 2016)	CD44 is a hyaluronan receptor mediating cell–matrix adhesion and extracellular matrix remodeling. The splice variant CD44v5 is upregulated in hypertrophied ligamentum flavum and enhances hyaluronan binding and fibrotic remodeling, whereas CD44v6 shows limited expression mainly around vascular structures without direct involvement in fibrosis.	CD44 can promote M2 polarization, whereas restricting hyaluronan–CD44 binding can shift toward M1 polarization in specific inflammatory contexts of tumor models. Among its variants, CD44v5 amplifies HA–CD44 signaling to induce M2-like macrophages, while CD44v6 has little evidence of modulating macrophage polarization.
angiogenic/proliferative	VEGF, Angopoietin-1, CD34^+^ microvessel density (Hur et al., 2015) (Moon et al., 2012; Jung et al., 2017; Takeuchi et al., 2016)	Angiogenic markers such as VEGF, Angiopoietin-1, and CD34^+^ microvessel density reflect endothelial proliferation and neovessel formation. In hypertrophied LF, VEGF and CD34^+^ expression are increased, indicating enhanced angiogenesis linking chronic inflammation to fibrotic remodeling, whereas Angiopoietin-1 contributes mainly to vascular stabilization.	VEGF promotes angiogenesis and fibrosis through M2 macrophage–associated remodeling in hypertrophic ligamentum flavum. Angiopoietin-1 enhances vascular stability and induces M2 macrophage polarization via Tie2 signaling. Increased CD34^+^ microvessel density reflects M2 macrophage–mediated angiogenesis and chronic fibrotic remodeling.
	PDGF-BB (Zhang et al., 2010) (Fang et al., 2024)	PDGF-BB is a fibroblast mitogen/chemoattractant linked to scar formation.	PDGF-BB regulates macrophage polarization context-dependently—enhancing M2-like activation in fibrotic and reparative tissues (kidney, bone, CNS), but correlating with M1-type inflammation in intestinal disease.
metabolic/endocrine	Leptin (Sun et al., 2018) (Li et al., 2025)	Leptin upregulates collagen I/III in LF cells by inducing IL-6 via the NF-κB pathway.	Leptin enhances M1 macrophage polarization in tendon-bone injury (rat model), indicating a pro-inflammatory macrophage bias in damaged tissue.
microRNA	miR-486-5p (Zhang et al., 2024) (Chang et al., 2020; Chai et al., 2019; Zhang et al., 2020)	miR-486-5p is an inflammation-related microRNA that correlates positively with IL-1β and TNF-α levels, suggesting its involvement in pro-inflammatory responses. Context-dependent pro- and anti-inflammatory effects have also been reported in other disease models.	In pro-inflammatory settings, miR-486-5p has been implicated in amplifying inflammatory signaling and may promote differentiation toward an M1 phenotype, although effects appear microenvironment-dependent.

Abbreviation: cluster of differentiation (CD), discoidin domain receptor (DDR), fractalkine (FKN), interleukin (IL), Janus kinase/signal transducer and activator of transcription (JAK/STAT), ligamentum flavum (LF), matrix metalloproteinase (MMP), monocyte chemoattractant protein-1 (MCP-1), nitric oxide metabolites (NOx), platelet-derived growth factor-BB (PDGF-BB), phosphorylated signal transducer and activator of transcription 3 (pSTAT3), vascular endothelial growth factor (VEGF).

### 3.4 Associations between biomarkers and clinical outcomes

NOx in CSF was negatively associated with postoperative recovery rate, indicating less favorable postoperative recovery, but no association with pre- or postoperative JOA scores (1 study, 70 patients, 53 controls). Higher serum MCP-1 was positively associated with short-term patient satisfaction following epidural steroid injection (1 study, 11 patients), suggesting a better perceived treatment response. In contrast, higher serum miR-486-5p levels were positively associated with pain and disability scores (VAS and ODI) and negatively associated with JOA scores at 6 months postoperatively, indicating worse clinical status; miR-486-5p levels was demonstrated to decrease after surgery (1 study, 52 patients, 42 controls). CSF IL-6 levels showed no association with pain scores or walking distance (1 study, 30 patients, 10 controls).

### 3.5 Associations between biomarkers and imaging features

Increased LF thickness was positively associated with tissue IL-6, p-STAT3, MMP-2, MMP-13, DDR1, DDR2, tissue CX3CL1/CX3CR1, soluble fractalkine (sFKN), CD44, CD44v5, PDGF-BB, leptin, VEGF, and microvessel density (CD34), but showed no association with CD44v6, MMP-9, angiopoietin-1 or VCAM. In addition, reduced dural sac CSA—reflecting more severe central stenosis—was associated with higher CSF IL-6, CD44, and CD44v5, but not with CD44v6. Overall, both LF hypertrophy and reduced dural sac CSA were associated with multiple biomarkers reported across the included studies.

## 4 Discussion

### 4.1 Clinical implications and future research

The available evidence on associations between inflammatory biomarkers and clinical outcomes in degenerative LSS remains scarce. This systematic review identified only a few biomarkers that were examined in relation to patient-centered outcomes. However, each of these associations was reported in a single study with low patient numbers, and their robustness and generalizability therefore remain uncertain. In contrast, the currently available literature is weighted toward associations with imaging severity. This imbalance helps define an important evidence gap for future biomarker research in degenerative LSS.

The current findings should be viewed as hypothesis-generating and suggest potential directions for future prognostic research in degenerative LSS: (i) evaluating whether NOx in CSF and miR-486-5p in serum are reproducibly associated with less favorable recovery, (ii) testing whether MCP-1 in serum identifies short-term responsiveness to conservative interventions such as epidural steroid injection, and (iii) determining whether peri-treatment biomarker changes in serum track symptom trajectories. Prospective studies with prespecified cut-offs, standardized outcomes, and appropriate adjustment for confounding are needed before these biomarkers can be considered for clinical use.

Notably, most biomarker–imaging associations were based on ligamentum flavum (LF) tissue obtained at surgery and are therefore not directly applicable to preoperative prognostic stratification. Accordingly, future prognostic research should prioritize biomarkers that can be measured before treatment (e.g., in serum or CSF) and assess how they complement imaging in predicting symptom trajectories and outcomes. At the same time, LF tissue remains a valuable resource for mechanistic studies: locally expressed biomarkers can help clarify inflammatory and immune processes in the spinal canal that may contribute to pain and disability beyond mechanical compression of neural elements.

### 4.2 Explaining clinical and image outcomes of biomarkers from the perspective of macrophage type I (M1) and type II (M2) polarization

In lumbar disc herniation, Djuric et al. (2020) summarized that pro-inflammatory mediators often discussed as M1-associated tend to correlate with worse pain-related outcomes. In contrast, anti-inflammatory mediators linked to M2-like responses have been hypothesized to be associated with more favorable clinical profiles, although findings are not uniform. In degenerative LSS, however, comparable evidence remains limited. Nevertheless, macrophage polarization may still provide a useful framework for considering the potential biological roles of candidate biomarkers identified in the present review.

Within this framework, elevated CSF NOx—reflecting iNOS-derived nitric oxide production by strongly activated M1 macrophages—may indicate a higher burden of M1-mediated neuroinflammation (Denda et al., 2011) (Rath et al., 2014). Consistent with this hypothesis, higher CSF NOx levels were associated with poorer postoperative recovery.

Higher baseline MCP-1, also known as CCL2 was described to reflect increased macrophage-recruitment and enhance environment dependent expression of M2 related cytokines (Lin et al., 2020) (Sierra-Filardi et al., 2014), potentially explaining its association with improved short-term satisfaction after epidural steroid injection; more broadly, glucocorticoids have been reported to suppress M1-associated programs and promote anti-inflammatory (M2-like) macrophage responses (Xie et al., 2019; Diaz-Jimenez et al., 2021).

In contrast, miR-486-5p is a relatively underexplored molecule with context-dependent inflammatory effects: two prior reports support a pro-inflammatory role, whereas one suggests anti-inflammatory effects (Chang et al., 2020; Chai et al., 2019; Zhang et al., 2020). In the study by Zhang et al. serum miR-486-5p levels correlated positively with TNF-α and IL-1β, indicating a pro-inflammatory milieu that may promote differentiation toward an M1 phenotype (Zhang et al., 2024). Accordingly, higher serum miR-486-5p levels were associated with greater pain and disability and poorer postoperative JOA scores, aligning with the conceptual framework proposed by Djuric et al. (2020)

**Fig. 2:**
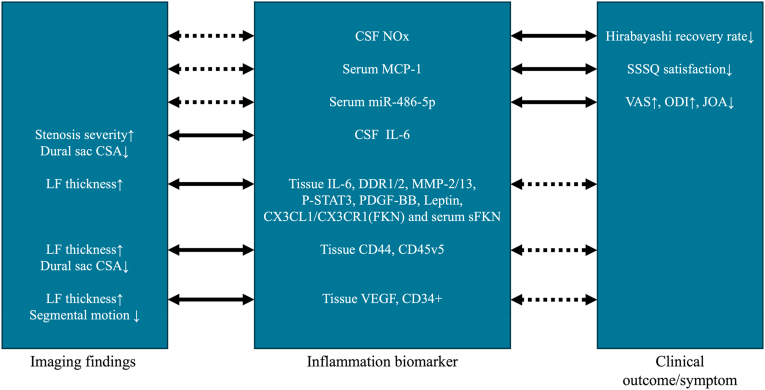
Most biomarkers were associated with either imaging findings or clinical outcomes (solid lines), while consistent evidence linking these two domains is lacking (dashed lines), highlighting the current evidence gap.

Most included articles focused on imaging outcomes, particularly LF hypertrophy and reduced dural sac CSA, and their associations with inflammatory, fibrotic, and angiogenic markers such as pSTAT3, VEGF, MMPs/DDR, and CX3CL1/CX3CR1. These findings suggest a link between inflammation and structural stenosis, but it is unclear to what extent such changes translate into more severe symptoms. In many studies, biomarker elevations and imaging abnormalities simply co-occur, without rigorous evaluation of whether they correspond to worse pain, disability, or function. As a result, the clinical meaning of these imaging–biomarker associations remains difficult to interpret (Fig. 2).

Imaging features such as LF hypertrophy and canal narrowing may reflect a predominantly M1-oriented inflammatory milieu driven by mediators such as IL-6 and CX3CL1, while markers influencing the balance between M1-and M2-like programs (e.g., STAT3, PDGF-BB, CD44/CD44v5, VEGF, and CD34^+^ microvessels) may contribute to remodeling and fibrosis (Jiang et al., 2024). Macrophage polarization is highly plastic and varies across organs and disease contexts (Murray, 2017). However, mechanistic data from lumbar spinal tissues and CSF in LSS remain sparse. Future studies are therefore needed to determine how the candidate biomarkers summarized in Table 2 map onto macrophage polarization states within the LSS microenvironment and how these profiles relate to symptoms and prognosis.

Direct evidence linking imaging severity and symptoms was limited to a single study in which higher CSF IL-6 levels were associated with reduced dural sac CSA but showed no correlation with pain intensity or walking distance. However, this study only concerned 30 patients and 10 controls. This limited evidence highlights the need for more comprehensive studies that integrate biomarkers, imaging, and standardized clinical outcomes to clarify how inflammatory activity contributes to the imaging–symptom gap in degenerative LSS and whether these biomarkers have prognostic utility.

### 4.3 Geographic distribution and generalizability

Most included studies were conducted in East Asia, which may limit generalizability to other populations. Given reported racial/ethnic differences in systemic inflammatory marker profiles (Wiley et al., 2025), the associations between inflammatory biomarkers, imaging features, and patient-centered outcomes should be interpreted with caution when extrapolating to other regions and ethnic groups.

### 4.4 Methodological limitations and future research

This review has several limitations, and the most important limitation is the small number of studies evaluating associations between inflammatory markers and clinical outcomes. No interventional studies were identified, precluding causal inference regarding treatment indications or interventions. Accordingly, trial designs that integrate preoperative screening and threshold-based treatment selection would be valuable to evaluate causal validity. In addition, heterogeneity in assay platforms and biospecimen processing reduced comparability of biomarker values, while insufficient multivariable adjustment left the possibility of residual confounding. Moreover, small sample sizes and variability in follow-up duration and assessment timing limited the precision of effect estimates and external validity. Finally, the predominance of East Asian studies raises concerns about the generalizability of the findings. Direct studies examining the relationship between inflammatory biomarkers and macrophage polarization in degenerative LSS are also scarce. Future research should focus on adequately powered prospective studies with standardized outcome measures and prespecified analytic plans, and should more directly investigate how inflammatory biomarkers relate to macrophage polarization, imaging findings, symptoms, and prognosis in degenerative LSS.

## 5 Conclusions

This systematic review highlights that evidence linking inflammatory biomarkers to patient-centered outcomes in degenerative LSS is scarce. Only NOx, MCP-1, and miR-486-5p were reported to be associated with clinical outcomes, each in a single study with a limited sample size, whereas most biomarker analyses focused on imaging features. Accordingly, these observations should be interpreted cautiously and considered hypothesis-generating. This imbalance highlights an important evidence gap and provides a basis for future prospective studies designed to clarify the clinical relevance of these biomarkers.

## Consent to participate

Not applicable.

## Author contributions

Michita Noma and Carmen Vleggeert-Lankamp designed the study; Carmen Vleggeert-Lankamp and Niek Djuric supervised the article; Michita Noma wrote the manuscript.

## Declarations ethical considerations

This study is a systematic review of published literature and did not involve human participants, patient data, or animals. Ethical approval and informed consent were not required.

## Consent for publication

Not applicable.

## Data availability statement

This study is a systematic review and did not generate new primary data. The datasets supporting the conclusions of this article, including detailed search strategies, study characteristics, and risk of bias assessments, are available in the article and its supplementary materials.

## Declaration of generative AI and AI-assisted technologies in the writing process

During the preparation of this work, the authors utilized ChatGPT-5 to enhance the readability and language of the manuscript. After employing this tool, the authors carefully reviewed and refined the content as necessary, and take full responsibility for the final publication.

## Funding statement

The authors received no financial support for the research, authorship, and/or publication of this article.

## Declaration of competing interest

The authors declare that they have no known competing financial interests or personal relationships that could have appeared to influence the work reported in this paper.
