# Supplementary material for: Associations of inflammatory biomarkers with clinical outcomes in degenerative lumbar spinal stenosis: A systematic review
Source: Brain Spine. 2026 Apr 18;6:106056. doi: 10.1016/j.bas.2026.106056 (PMC13125152; doi:10.1016/j.bas.2026.106056)
Supplement: Multimedia component 1 [file mmc1.doc]

# **Supplementary File: Full Electronic Search Strategies**

Databases searched: MEDLINE/PubMed, Embase, Web of Science Core Collection, Cochrane Library, CINAHL, Academic Search Premier.
Time coverage: Inception to 4 July 2025.
Search concepts: (1) Lumbar spinal stenosis, (2) Inflammation/immune mediators, (3) Humans; with animal-only records and conference abstracts excluded where database functionality allowed.
De-duplication: Results were exported to a reference manager and de-duplicated prior to screening; the deduplicated set was then screened in Rayyan (RIS). Totals on 4 July 2025: 420 unique records after de-duplication (database-level yields shown below).

Total 420 references, sourced from:

- PubMed: 160
- Embase: 266 - 189 unique (including 42 trial-registry records)
- Web of Science: 134 - 44 unique
- Cochrane Library: 28 - 21 unique (including 13 trial-registry records)
- CINAHL: 16 - 3 unique
- Academic Search Premier: 41 - 3 unique

**Databases:**

**PubMed**

(("lumbar stenosis"[tw] OR "lumbar stenoses"[tw] OR "lumbar stenosis"[tiab:~6] OR "lumbar stenoses"[tiab:~6] OR "lumbal stenosis"[tiab:~6] OR "lumbal stenoses"[tiab:~6] OR (("Spinal Stenosis"[mesh] OR "spinal stenosis"[tw] OR "spinal stenoses"[tw]) AND ("Lumbar Vertebrae"[mesh])) OR (("stenosis"[ti] OR "stenoses"[ti]) AND ("Lumbar"[ti] OR "lumbal"[ti]))) AND ("Inflammation"[mesh] OR "Inflammation"[tw] OR "Inflammations"[tw] OR "Neurogenic Inflammation"[mesh] OR "Neurogenic Inflammation"[tw] OR "Inflammation Mediators"[mesh] OR "Macrophages"[tw] OR "Macrophage"[tw] OR "Macrophages"[mesh] OR "Leukocytes"[mesh] OR "Leukocytes"[tw] OR "leukocyte"[tw] OR "Leukocytes, Mononuclear"[mesh] OR "Cytokine-Induced Killer Cells"[tw] OR "Cytokine-Induced Killer Cells"[mesh] OR "Lymphocytes"[tw] OR "Lymphocyte"[tw] OR "Lymphocytes"[mesh]) NOT (("Animals"[mesh] OR **"veterinary"[ti] OR "rabbit"[ti] OR "rabbits"[ti] OR "animal"[ti] OR "animals"[ti] OR "murine"[ti] OR "mouse"[ti] OR "mice"[ti] OR "rodent"[ti] OR "rodents"[ti] OR "rat"[ti] OR "rats"[ti] OR "pig"[ti] OR "pigs"[ti] OR "porcine"[ti] OR "horse"[ti] OR "horses"[ti] OR "equine"[ti] OR "cow"[ti] OR "cows"[ti] OR "bovine"[ti] OR "goat"[ti] OR "goats"[ti] OR "sheep"[ti] OR "ovine"[ti] OR "canine"[ti] OR "dog"[ti] OR "dogs"[ti] OR "feline"[ti] OR "cat"[ti] OR "cats"[ti]) NOT "Humans"[mesh]))**

**Embase**

**(**((exp *"lumbar spinal stenosis"/ OR "lumbar stenosis".ti,ab OR "lumbar stenoses".ti,ab OR ("lumbar" ADJ6 "stenosis").ti,ab OR ("lumbar" ADJ6 "stenoses").ti,ab OR ("lumbal" ADJ6 "stenosis").ti,ab OR ("lumbal" ADJ6 "stenoses").ti,ab OR ((exp *"Vertebral canal Stenosis"/ OR "spinal stenosis".ti,ab OR "spinal stenoses".ti,ab) AND (exp *"Lumbar Spine"/)) OR (("stenosis".ti OR "stenoses".ti) AND ("Lumbar".ti OR "lumbal".ti))) AND (*"Inflammation"/ OR "Inflammation".ti,ab OR "Inflammations".ti,ab OR exp *"Neurogenic Inflammation"/ OR "Neurogenic Inflammation".ti,ab OR exp *"autacoid"/ OR "Macrophages".ti,ab OR "Macrophage".ti,ab OR exp *"Macrophage"/ OR exp *"Leukocyte"/ OR "Leukocytes".ti,ab OR "leukocyte".ti,ab OR exp *"Mononuclear Cell"/ OR "Cytokine-Induced Killer Cells".ti,ab OR exp *"Cytokine Induced Killer Cell"/ OR "Lymphocytes".ti,ab OR "Lymphocyte".ti,ab OR exp *"Lymphocyte"/) NOT ((exp "Animals"/ OR **"veterinary".ti OR "rabbit".ti OR "rabbits".ti OR "animal".ti OR "animals".ti OR "murine".ti OR "mouse".ti OR "mice".ti OR "rodent".ti OR "rodents".ti OR "rat".ti OR "rats".ti OR "pig".ti OR "pigs".ti OR "porcine".ti OR "horse".ti OR "horses".ti OR "equine".ti OR "cow".ti OR "cows".ti OR "bovine".ti OR "goat".ti OR "goats".ti OR "sheep".ti OR "ovine".ti OR "canine".ti OR "dog".ti OR "dogs".ti OR "feline".ti OR "cat".ti OR "cats".ti) NOT exp "Humans"/)** **NOT (conference review or conference abstract).pt) OR** ((exp *"lumbar spinal stenosis"/ OR "lumbar stenosis".ti OR "lumbar stenoses".ti OR ("lumbar" ADJ6 "stenosis").ti OR ("lumbar" ADJ6 "stenoses").ti OR ("lumbal" ADJ6 "stenosis").ti OR ("lumbal" ADJ6 "stenoses").ti OR ((exp *"Vertebral canal Stenosis"/ OR "spinal stenosis".ti OR "spinal stenoses".ti) AND (exp *"Lumbar Spine"/)) OR (("stenosis".ti OR "stenoses".ti) AND ("Lumbar".ti OR "lumbal".ti))) AND (exp *"Inflammation"/) NOT ((exp "Animals"/ OR **"veterinary".ti OR "rabbit".ti OR "rabbits".ti OR "animal".ti OR "animals".ti OR "murine".ti OR "mouse".ti OR "mice".ti OR "rodent".ti OR "rodents".ti OR "rat".ti OR "rats".ti OR "pig".ti OR "pigs".ti OR "porcine".ti OR "horse".ti OR "horses".ti OR "equine".ti OR "cow".ti OR "cows".ti OR "bovine".ti OR "goat".ti OR "goats".ti OR "sheep".ti OR "ovine".ti OR "canine".ti OR "dog".ti OR "dogs".ti OR "feline".ti OR "cat".ti OR "cats".ti) NOT exp "Humans"/)** **NOT (conference review or conference abstract).pt))**

**Web of Science**

((TS=("lumbar spinal stenosis" OR "lumbar stenosis" OR "lumbar stenoses" OR ("lumbar" NEAR/6 "stenosis") OR ("lumbar" NEAR/6 "stenoses") OR ("lumbal" NEAR/6 "stenosis") OR ("lumbal" NEAR/6 "stenoses") OR (("Vertebral canal Stenosis" OR "spinal stenosis" OR "spinal stenoses") AND ("Lumbar Spine"))) OR TI=(("stenosis" OR "stenoses") AND ("Lumbar" OR "lumbal"))) AND TS=("Inflammation" OR "Inflammation" OR "Inflammations" OR "Neurogenic Inflammation" OR "Neurogenic Inflammation" OR "autacoid" OR "Macrophages" OR "Macrophage" OR "Macrophage" OR "Leukocyte" OR "Leukocytes" OR "leukocyte" OR "Mononuclear Cell" OR "Cytokine-Induced Killer Cells" OR "Cytokine Induced Killer Cell" OR "Lymphocytes" OR "Lymphocyte" OR "Lymphocyte") **NOT (TI=("veterinary" OR "rabbit" OR "rabbits" OR "animal" OR "animals" OR "murine" OR "mouse" OR "mice" OR "rodent" OR "rodents" OR "rat" OR "rats" OR "pig" OR "pigs" OR "porcine" OR "horse" OR "horses" OR "equine" OR "cow" OR "cows" OR "bovine" OR "goat" OR "goats" OR "sheep" OR "ovine" OR "canine" OR "dog" OR "dogs" OR "feline" OR "cat" OR "cats") OR AK=("veterinary" OR "rabbit" OR "rabbits" OR "animal" OR "animals" OR "murine" OR "mouse" OR "mice" OR "rodent" OR "rodents" OR "rat" OR "rats" OR "pig" OR "pigs" OR "porcine" OR "horse" OR "horses" OR "equine" OR "cow" OR "cows" OR "bovine" OR "goat" OR "goats" OR "sheep" OR "ovine" OR "canine" OR "dog" OR "dogs" OR "feline" OR "cat" OR "cats")) NOT DT=(meeting abstract))**

**Cochrane Library**

((("lumbar spinal stenosis" OR "lumbar stenosis" OR "lumbar stenoses" OR ("lumbar" NEAR/6 "stenosis") OR ("lumbar" NEAR/6 "stenoses") OR ("lumbal" NEAR/6 "stenosis") OR ("lumbal" NEAR/6 "stenoses") OR (("Vertebral canal Stenosis" OR "spinal stenosis" OR "spinal stenoses") AND ("Lumbar Spine"))):ti,ab,kw OR (("stenosis" OR "stenoses") AND ("Lumbar" OR "lumbal")):ti) AND ("Inflammation" OR "Inflammation" OR "Inflammations" OR "Neurogenic Inflammation" OR "Neurogenic Inflammation" OR "autacoid" OR "Macrophages" OR "Macrophage" OR "Macrophage" OR "Leukocyte" OR "Leukocytes" OR "leukocyte" OR "Mononuclear Cell" OR "Cytokine-Induced Killer Cells" OR "Cytokine Induced Killer Cell" OR "Lymphocytes" OR "Lymphocyte" OR "Lymphocyte"):ti,ab,kw)

**CINAHL**

((TX("lumbar spinal stenosis" OR "lumbar stenosis" OR "lumbar stenoses" OR ("lumbar" NEAR/6 "stenosis") OR ("lumbar" NEAR/6 "stenoses") OR ("lumbal" NEAR/6 "stenosis") OR ("lumbal" NEAR/6 "stenoses") OR (("Vertebral canal Stenosis" OR "spinal stenosis" OR "spinal stenoses") AND ("Lumbar Spine"))) OR TI(("stenosis" OR "stenoses") AND ("Lumbar" OR "lumbal"))) AND TX("Inflammation" OR "Inflammation" OR "Inflammations" OR "Neurogenic Inflammation" OR "Neurogenic Inflammation" OR "autacoid" OR "Macrophages" OR "Macrophage" OR "Macrophage" OR "Leukocyte" OR "Leukocytes" OR "leukocyte" OR "Mononuclear Cell" OR "Cytokine-Induced Killer Cells" OR "Cytokine Induced Killer Cell" OR "Lymphocytes" OR "Lymphocyte" OR "Lymphocyte") **NOT (TI("veterinary" OR "rabbit" OR "rabbits" OR "animal" OR "animals" OR "murine" OR "mouse" OR "mice" OR "rodent" OR "rodents" OR "rat" OR "rats" OR "pig" OR "pigs" OR "porcine" OR "horse" OR "horses" OR "equine" OR "cow" OR "cows" OR "bovine" OR "goat" OR "goats" OR "sheep" OR "ovine" OR "canine" OR "dog" OR "dogs" OR "feline" OR "cat" OR "cats") OR MW("veterinary" OR "rabbit" OR "rabbits" OR "animal" OR "animals" OR "murine" OR "mouse" OR "mice" OR "rodent" OR "rodents" OR "rat" OR "rats" OR "pig" OR "pigs" OR "porcine" OR "horse" OR "horses" OR "equine" OR "cow" OR "cows" OR "bovine" OR "goat" OR "goats" OR "sheep" OR "ovine" OR "canine" OR "dog" OR "dogs" OR "feline" OR "cat" OR "cats")))**

**Academic Search Premier**

((XB("lumbar spinal stenosis" OR "lumbar stenosis" OR "lumbar stenoses" OR ("lumbar" NEAR/6 "stenosis") OR ("lumbar" NEAR/6 "stenoses") OR ("lumbal" NEAR/6 "stenosis") OR ("lumbal" NEAR/6 "stenoses") OR (("Vertebral canal Stenosis" OR "spinal stenosis" OR "spinal stenoses") AND ("Lumbar Spine"))) OR TI(("stenosis" OR "stenoses") AND ("Lumbar" OR "lumbal"))) AND XB("Inflammation" OR "Inflammation" OR "Inflammations" OR "Neurogenic Inflammation" OR "Neurogenic Inflammation" OR "autacoid" OR "Macrophages" OR "Macrophage" OR "Macrophage" OR "Leukocyte" OR "Leukocytes" OR "leukocyte" OR "Mononuclear Cell" OR "Cytokine-Induced Killer Cells" OR "Cytokine Induced Killer Cell" OR "Lymphocytes" OR "Lymphocyte" OR "Lymphocyte") **NOT (TI("veterinary" OR "rabbit" OR "rabbits" OR "animal" OR "animals" OR "murine" OR "mouse" OR "mice" OR "rodent" OR "rodents" OR "rat" OR "rats" OR "pig" OR "pigs" OR "porcine" OR "horse" OR "horses" OR "equine" OR "cow" OR "cows" OR "bovine" OR "goat" OR "goats" OR "sheep" OR "ovine" OR "canine" OR "dog" OR "dogs" OR "feline" OR "cat" OR "cats") OR SU("veterinary" OR "rabbit" OR "rabbits" OR "animal" OR "animals" OR "murine" OR "mouse" OR "mice" OR "rodent" OR "rodents" OR "rat" OR "rats" OR "pig" OR "pigs" OR "porcine" OR "horse" OR "horses" OR "equine" OR "cow" OR "cows" OR "bovine" OR "goat" OR "goats" OR "sheep" OR "ovine" OR "canine" OR "dog" OR "dogs" OR "feline" OR "cat" OR "cats")))**
